# A *RAB3GAP1* SINE Insertion in Alaskan Huskies with Polyneuropathy, Ocular Abnormalities, and Neuronal Vacuolation (POANV) Resembling Human Warburg Micro Syndrome 1 (WARBM1)

**DOI:** 10.1534/g3.115.022707

**Published:** 2015-11-23

**Authors:** Michaela Wiedmer, Anna Oevermann, Stephanie E. Borer-Germann, Daniela Gorgas, G. Diane Shelton, Michaela Drögemüller, Vidhya Jagannathan, Diana Henke, Tosso Leeb

**Affiliations:** * Institute of Genetics, Vetsuisse Faculty, University of Bern, 3001 Bern, Switzerland; † Division of Neurological Sciences, Department of Clinical Veterinary Medicine, Vetsuisse Faculty, University of Bern, 3001 Bern, Switzerland; ‡ Ophthalmology Service, Department of Clinical Veterinary Medicine, Vetsuisse Faculty, University of Bern, 3001 Bern, Switzerland; § Division of Clinical Radiology, Department of Clinical Veterinary Medicine, Vetsuisse Faculty, University of Bern, 3001 Bern, Switzerland; ** Department of Pathology, School of Medicine, University of California San Diego, La Jolla, California 92093

**Keywords:** dog, *canis familiaris*, whole genome sequencing, animal model, linkage, homozygosity

## Abstract

We observed a hereditary phenotype in Alaskan Huskies that was characterized by polyneuropathy with ocular abnormalities and neuronal vacuolation (POANV). The affected dogs developed a progressive severe ataxia, which led to euthanasia between 8 and 16 months of age. The pedigrees were consistent with a monogenic autosomal recessive inheritance. We localized the causative genetic defect to a 4 Mb interval on chromosome 19 by a combined linkage and homozygosity mapping approach. Whole genome sequencing of one affected dog, an obligate carrier, and an unrelated control revealed a 218-bp SINE insertion into exon 7 of the *RAB3GAP1* gene. The SINE insertion was perfectly associated with the disease phenotype in a cohort of 43 Alaskan Huskies, and it was absent from 541 control dogs of diverse other breeds. The SINE insertion induced aberrant splicing and led to a transcript with a greatly altered exon 7. *RAB3GAP1* loss-of-function variants in humans cause Warburg Micro Syndrome 1 (WARBM1), which is characterized by additional developmental defects compared to canine POANV, whereas *Rab3gap1*-deficient mice have a much milder phenotype than either humans or dogs. Thus, the *RAB3GAP1* mutant Alaskan Huskies provide an interesting intermediate phenotype that may help to better understand the function of *RAB3GAP1* in development. Furthermore, the identification of the presumed causative genetic variant will enable genetic testing to avoid the nonintentional breeding of affected dogs.

Warburg Micro Syndrome (WARBM), also known as Micro Syndrome, is a severe rare disorder with an autosomal recessive inheritance in humans. The clinical phenotype is characterized by micropthalmia, microcornea, bilateral congenital cataracts, short palpebral fissures, optic atrophy, severe mental retardation, congenital hypotonia with subsequent spasticity, and variable toe malformations. Brain magnetic resonance imaging (MRI) findings show relatively consistent patterns of delayed myelination, polymicrogyria of the frontal and parietal lobes, wide sylvian fissures, corpus callosum hypogenesis, and increased subdural spaces (Warburg *et al.* 1993; Morris-Rosendahl *et al.* 2010).

WARBM is a heterogenetic disorder in humans, and variants in several genes lead to clinically indistinguishable phenotypes (Liegel *et al.* 2013). WARBM1 (MIM 600118) is caused by variants in the *RAB3GAP1* gene encoding the catalytic RAB3 GTPase-activating protein subunit 1 (Aligianis *et al.* 2005). WARBM2 (MIM 614265), WARBM3 (MIM 614222), and WARBM4 (MIM 615663) are caused by genetic variants in *RAB3GAP2*, *RAB18*, and *TBC1D20*, respectively. Large 1q43-44 deletions may also result in a WARBM phenotype (Arroyo-Carrera *et al.* 2015).

RAB3GAP1 is a 130-kDa protein that forms, together with the 150 kDa RAB3GAP2, the heterodimeric RAB3GAP complex. This complex regulates the activity of members of the RAB3 subfamily of small G proteins belonging to the RAS superfamily. RAB proteins are master regulators of intracellular vesicle trafficking. They regulate the budding of transport vesicles from the donor membrane, motility, docking, and fusion with the acceptor membrane (Fukui *et al.* 1997; Oishi *et al.* 1998; Handley *et al.* 2013). RAB family members cycle between a GDP-bound inactive and a GTP-bound active form. The GTP-bound active form of RAB3 family members is inactivated by GTP hydrolysis before, during, and after the fusion of the vesicle by the stimulation of RAB3GAP (Fukui *et al.* 1997). RAB3 family members have their function in regulated exocytosis of hormones and neurotransmitters, and RAB3A is located in synaptic vesicles and secretory granules (Oishi *et al.* 1998; Handley *et al.* 2013).

WARBM1 causative variants in the *RAB3GAP1* gene are thought to severely affect the catalytic activity of *RAB3GAP1* enzyme function and/or to provoke nonsense-mediated decay of the *RAB3GAP1* message (loss-of-function mutations). Less damaging variants are suggested to cause the milder Martsolf syndrome (Aligianis *et al.* 2005; Handley *et al.* 2013).

In this report, we describe Alaskan Huskies with neurological and ocular abnormalities with some parallels to the human WARBM phenotype. Several affected dogs were clinically and histopathologically characterized. The main focus of this study was the identification of the presumed causative genetic defect by a positional cloning approach.

## Materials and Methods

### Ethics statement

All animal experiments were performed according to the local regulations. All dogs in this study were privately owned, and examined with the consent of their owners. The collection of blood samples was approved by the “Cantonal Committee For Animal Experiments” (Canton of Bern; permit 23/10).

### Breed nomenclature

The neurological defect was observed in Alaskan Huskies. This term is used for working dogs that are bred and used for sled racing. Alaskan Huskies are not recognized as a breed by the American Kennel Club, or other national kennel clubs. They represent an admixed population with contributions from Siberian Huskies, Alaskan Malamutes, local dogs bred by the Inuit and other inhabitants of the polar regions, and some other purebred dogs deemed suitable for sled pulling. In contrast, Siberian Huskies and Alaskan Malamutes are recognized breeds, and represent closed populations.

### Clinical examinations

We performed clinical examinations on 33 Alaskan Huskies at the Small Animal Hospital of the University of Bern between 2012 and 2015. These consisted of six affected and 27 clinically unremarkable dogs. General clinical, neurological, and ophthalmologic examinations were performed, and gender, age at onset of clinical signs, the course of the disease, and age at time of euthanasia were recorded. In selected affected dogs, further diagnostic tests, such as blood work (*n* = 3), urinalysis (*n* = 3), cerebrospinal fluid (CSF) analysis (*n* = 2), radiographs of chest (*n* = 3), electrodiagnostics (*n* = 2), muscle and nerve biopsies (*n* = 3), and magnetic resonance imaging (*n* = 4) were performed. MRI was performed in four affected, one age-matched clear, and one carrier Alaskan Husky. Examinations of the head (*n* = 6), and thoracolumbar spine (*n* = 5) were performed in either a low-field magnet (0.3 T; *n* = 1) or a high-field magnet (*n* = 5). One affected dog underwent two examinations, the first at the age of 13 months and the second one 3 months later directly before euthanasia. MRI evaluation included subjective assessment of external (CSF) spaces, assessment of cerebral white matter and corpus callosum thickness, shape of the thoracolumbar spinal cord, and size evaluated as ratio of the spinal cord area to the vertebral canal area in transverse direction at the level of the 13th thoracic vertebra.

### Neuropathology and immunohistochemistry

The brain, spinal cord and nerves were examined from five affected Huskies after euthanasia (SY001, male, 16 months; SY006, female, 8 months; SY030, female, 8 months; SY041, male, 11 months; SY042 female, 11 months). Samples were immersion-fixed in 4% neutral-buffered formaldehyde. Representative tissue samples were processed, embedded in paraffin, sectioned at 5 μm and stained with hematoxylin and eosin (HE). Selected brain and spinal cord sections were stained with Luxol Fast Blue and HE (LFB-HE), or with Bielschowsky silver stain. Additionally, immunohistochemistry was performed with the monoclonal L42 antibody (R-Biopharm, Darmstadt, Germany) as previously described to exclude pathological Prion protein (PrP^d^) deposition (Seuberlich *et al.* 2007).

Additionally, nerve and muscle biopsies were collected from three affected dogs (SY001, SY041, SY042) at 11–12 months of age. Fresh and fixed biopsies from the cranial tibial muscle, and from the peroneal, vagus, and vagosympathetic nerves were shipped under refrigeration by an express service to the Comparative Neuromuscular Laboratory, University of California San Diego (La Jolla, CA). Upon receipt, tissues were frozen in isopentane precooled in liquid nitrogen and stored at –80° until further processed. Cryosections (8 µm) were stained or reacted with HE, modified Gomori trichrome, periodic acid-Schiff (PAS), myofibrillar ATPases at pH 9.8 and 4.3, esterase, nicotinamide adenine dinucleotide-tetrazolium reductase (NADH-TR), succinate dehydrogenase (SDH), cytochrome *c* oxidase, acid phosphatase, alkaline phosphatase, oil red O, and staphylococcal protein A conjugated to horseradish peroxidase (SPA-HRPO), by standard protocols (Dubowitz and Sewry 2013). In addition, nerve specimens were fixed in 10% neutral buffered formalin, resin-embedded, then evaluated in 1 µm plastic sections stained with toluidine blue.

### Animals for the genetic analysis

We used 43 Alaskan Huskies for the genetic analysis; 41 of these dogs originated from a single breeder. The Alaskan Huskies included the 33 dogs that were examined by clinical neurologists (six cases/27 controls), and 10 additional control dogs that were reported as unremarkable by their owners. We additionally investigated two purebred Siberian Huskies, six Alaskan Malamutes, 25 Samoyedes, 15 Greenland Dogs, and 493 dogs from 64 various diverse dog breeds that had been donated to the biobank of the Institute of Genetics at the University of Bern. The 541 purebred dogs are listed in detail in Supporting Information, Table S1.

It should be noted that we continued to collect samples during this study. Therefore, we started with smaller sample numbers during the genetic mapping, and only used the full number of samples for genotyping the SINE insertion for the final association analysis.

### DNA samples and single nucleotide polymorphism (SNP) genotyping

We isolated genomic DNA from EDTA blood samples with the Nucleon Bacc2 kit (GE Healthcare). Genotyping was done on Illumina canine_HD chips containing 173,662 SNPs by GeneSeek/Neogen. Genotypes were stored in a BC/Gene database version 3.5 (BC/Platforms).

### Linkage and homozygosity mapping

For the linkage analysis we had Illumina canine_HD SNP chip genotypes from 18 dogs, four parents, and 14 offspring (three affected/11 nonaffected, Figure S1). We removed noninformative markers, markers on the sex chromosomes, and markers that were not genotyped in all animals. The pruned dataset contained 64,358 markers. We then applied the Merlin software (Abecasis *et al.* 2002), and a fully penetrant, recessive model of inheritance to analyze the data for parametric linkage.

We used PLINK v1.07 (Purcell *et al.* 2007) to search for extended intervals of homozygosity with shared alleles across four affected animals. The options –homozyg and –homozyg-group were applied. Using these standard parameters, PLINK reports homozygous segments ≥1 Mb. The final definition of the minimal critical interval was done by visual inspection of all SNP chip genotypes of the four genotyped cases on chromosome 19 in an Excel file.

### Gene analysis

We used the dog CanFam 3.1 assembly for all analyses. All numbering within the canine *RAB3GAP1* gene corresponds to the accession XM_851254.3 (mRNA).

### Whole genome sequencing of three dog genomes

We prepared PCR-free fragment libraries with 300 bp insert sizes from an affected Alaskan Husky (SY001), an obligate carrier Alaskan Husky (SY018), and a nonaffected unrelated purebred Siberian Husky. The libraries were sequenced to 13x–19x coverage on an Illumina HiSeq2500 instrument using 2 × 100 bp paired-end reads. The mapping and variant calling was done as described previously (Drögemüller *et al.* 2014). The reads obtained from whole genome sequencing were also visualized in the Integrative Genomics Viewer (IGV) (Broad Institute).

### PCR and Sanger sequencing

We used Sanger sequencing to confirm variants identified from whole genome sequencing. For these experiments, we amplified PCR products from genomic DNA using SequalPrep long-range polymerase (Thermo Fisher). The PCR primers used for the genotyping of the SINE insertion were GTCCATTCCCATTTAATTGTGTCCT and AGAGGAAAAGGAGTAGGAAGAGA (regular nonmodified primers). PCR products were directly sequenced on an ABI 3730 capillary sequencer (Thermo Fisher) after treatment with exonuclease I, and shrimp alkaline phosphatase. We analyzed the Sanger sequence data with Sequencher 5.1 (GeneCodes).

### Fragment length analysis

We used fragment length analyses to genotype a larger number of samples. The size of the PCR products was determined on the Fragment Analyzer capillary gel electrophoresis instrument (Advanced Analytical). Visual inspection of the output file prompted us to classify the dogs as homozygous for the SINE insertion (ins/ins, single band of ∼690 bp), heterozygous (ins/wt, two bands of 472 and ∼690 bp) or homozygous wildtype (wt/wt, single band of 472 bp).

### RNA isolation and RT-PCR

We isolated total RNA from brain using Qiazol and RNeasy spin columns according to the manufacturer’s recommendations (Qiagen). Total RNA from blood was isolated using PAXgene tubes, and the PAXgene blood RNA kit (Qiagen). All RNA samples were treated with RNase-free DNase to remove contaminations with genomic DNA. Reverse transcription to generate cDNA was carried out using an oligo-dT primer, and Superscript IV reverse transcriptase according to the manufacturer’s recommendations (Thermo Fisher). We performed RT-PCR with 1 µl of the synthesized cDNA, and SequalPrep long range polymerase (Thermo Fisher). Primers located in exon 6 and exon 8 of the *RAB3GAP1* gene were CCAAGAGCACATTGCCTGGT and CAGGCCTCCAACTTCTCCTC. RT-PCR products were directly Sanger sequenced as described above.

### Data availability

File S1 is a video illustrating the clinical phenotype of an affected Alaskan Husky (SY005) at 8 months of age. File S2 lists the sequence context of the 218 bp SINE insertion into exon 7 of the canine *RAB3GAP1* gene. File S3 shows an alignment of the canine wildtype and predicted mutant RAB3GAP1 protein. Figure S1 shows the pedigrees of Alaskan Huskies used for the mapping of the disease locus. Figure S2 represents an IGV screenshot of the region with the SINE insertion. Table S1 contains *RAB3GAP1:c.614_615ins218* genotypes of 541 dogs from 68 different dog breeds. Table S2 lists genome regions that showed positive LOD scores in the linkage analysis. The raw SNP chip genotypes are available upon request. The genome sequencing data were deposited in the European Nucleotide Archive (ENA) under accessions PRJEB9590 (case), PRJEB9591 (carrier), PRJEB10823 (control). The nucleotide sequence of the mutant *RAB3GAP1* allele with the SINE insertion was deposited in the ENA under accession LN864704.

## Results

### Clinical presentation and laboratory findings

Of the examined dogs, four female and two male dogs displayed an abnormal neurological examination. The neurological signs started at the age of 4–5 months with visual problems, and 1–2 months later the dogs displayed an altered voice, regurgitation, and gait abnormalities progressing to a severe ataxia within another 2–6 months. A video of an affected dog is presented as File S1.

**Figure 1:**
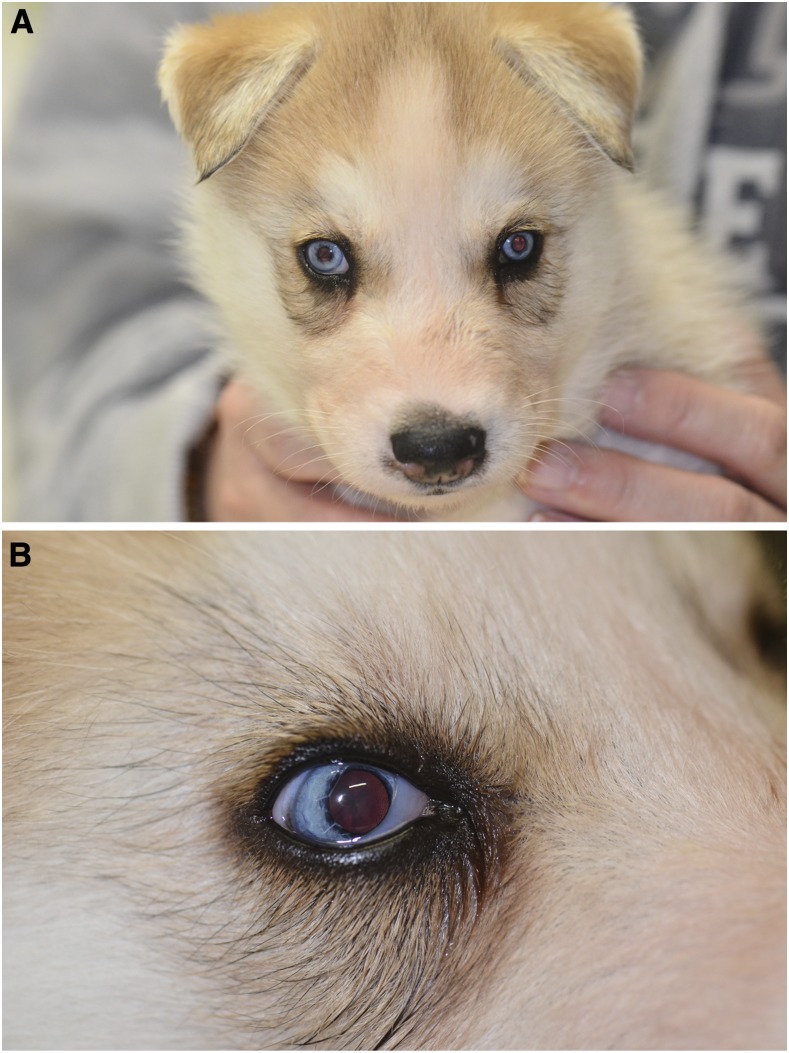
Eye phenotype in a 5-week-old affected Alaskan Husky. Multiple ocular anomalies such as (A) microphthalmia and (B) persistent pupillary membranes and an immature cataract can be noted.

On presentation at the age of 6–10 months, the general physical examination revealed no abnormalities. Consciousness was evaluated as normal during the neurological examination. However, the dogs were very anxious during manipulating the head. The dogs had a severe spinal ataxia, with the pelvic limbs being more severely affected than the thoracic limbs, and a mild tetraparesis. Postural reactions were absent in the pelvic limbs, and mildly reduced in the thoracic limbs. The menace response was absent bilaterally, and other cranial nerves were normal. The segmental spinal reflexes of all four limbs were normal to increased. Two dogs had a crossed extensor-flexor reflex in both pelvic limbs, and a severely increased muscle tone. The dogs were not painful on palpation of the spine (File S1). Ophthalmologic examination revealed bilateral microphthalmia, small pupils, and lenses with nuclear cataract (Figure 1). Four of the affected dogs additionally exhibited strabismus and/or persistent pupillary membranes.

The lesions were neuroanatomically localized to the spinal cord (sensory > motor), the eyes, and vagus nerve. Blood work, urinalysis, and CSF examination were unremarkable. Radiographs of the chest revealed a megaesophagus in all examined dogs. Electrodiagnostic testing including electromyography, measurement of sensory and motor nerve conduction velocities, and measurement of the compound muscle action potential following repetitive nerve stimulation was performed in two affected dogs at the age of 11 and 13 months under general anesthesia. The only abnormalities identified were decremental responses of 20% of the compound muscle action potential following repetitive nerve stimulation of the peroneal nerve (stimulation frequency: 2 Hz; stimulation duration 0.1 msec).

### MRI findings

The sulci of the forebrain were subjectively wide in all six dogs that underwent MRI, with mild enlargement in the nonaffected and one affected dog. In the remaining four dogs, there was moderate (three affected) to marked (one affected, one carrier) enlargement. The white matter of the forebrain and corpus callosum was thinner in all affected dogs compared to the carrier and clear dog. Except for the clear dog, external CSF spaces within the caudal fossa and cerebellar fissures were enlarged, with mild enlargement in three dogs, moderate enlargement in two (including the carrier dog), and marked enlargement in one dog. The thoracolumbar spinal cord was triangular with thinning of the dorsal parts in three affected dogs. The ratio between spinal cord area to the spinal canal area was smaller in all affected dogs (≤ 0.19) compared to the carrier and clear dog (≥ 0.2). In the one affected dog with two examinations, there was no visible difference between the findings of both examinations.

### Neuropathological findings

**Figure 2:**
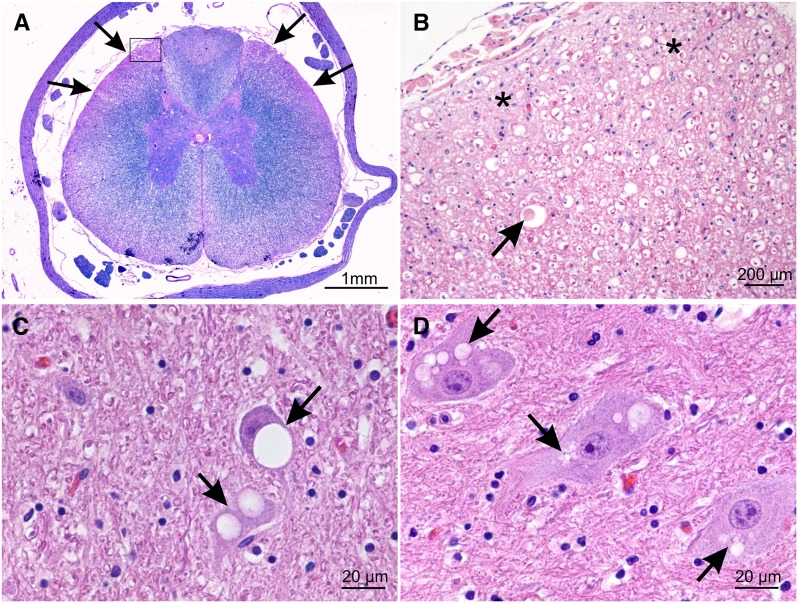
Neuropathologic findings in the central nervous system of the affected Alaskan Husky SY001. Lesions are consistent with neuronal vacuolation and spinocerebellar degeneration. (A) Combined LFB-HE stain of a thoracic spinal cord cross-section. Bilateral-symmetrical axonal and myelin loss, characterized by loss of blue color, is most prominent in the superficial dorsolateral tracts (arrows). (B) Higher magnification of the dorsolateral tract indicated by the rectangle in A. Myelin sheets are multifocally dilated, and some contain axonal spheroids (arrow) and myelinophages. The white matter is replaced by gliotic tissue (asterisks), characterized by a paler staining and increased number of astrocytic nuclei. (C) Nucleus interpositus. Two neurons contain large, clearly defined vacuoles (arrows). The nucleus of the upper neurons is displaced to the periphery by the intracytoplasmic vacuole. (D) Nucleus interpositus. Three neurons with multiple coalescing vacuoles (arrows) in the cytoplasm.

Neuropathological examinations were performed on five affected Alaskan Huskies, and showed bilaterally symmetrical chronic Wallerian-type axonal degeneration in the spinal cord, which was characterized by dilated myelin sheaths containing either axonal spheroids and fragments or myelinophages. Lesions were most prominent in the superficial dorsolateral white matter tracts of the cervical and thoracic segments (Figure 2A), where they consisted of areas of axonal and myelin loss replaced by gliotic tissue (Figure 2B). Additionally, widely spread, bilateral-symmetrical, subtle-to-severe neuronal vacuolation was present in the spinal cord gray matter, facial nucleus, gracile and cuneate nuclei, vestibular nuclei, cerebellar nuclei, oculomotor nuclei, substantia nigra, thalamic nuclei, hypothalamus, hippocampus, and cortex. The vacuolation was characterized by the presence of one to multiple clearly defined vacuoles of varying size in the neuronal somata, and was prominent in the cerebellar nuclei (Figure 2, C and D). Vacuoles were also observed in the surrounding neuropil, which contained scattered axonal spheroids, and was gliotic. In the cerebellar cortex, mild-to-severe Purkinje cell degeneration and loss were observed, associated with cerebellar atrophy in one case. Scattered axonal spheroids were present in the granule cell layer. Mild vacuolation and scattered fragmented axons were observed in the white matter of the cerebellum and brainstem. Pathological prion protein deposition was absent.

In muscle and peripheral nerve biopsies from three affected dogs, we observed a mild variability in myofiber size, with scattered atrophic fibers having an angular to anguloid shape, and of both fiber types. Multifocal areas of type 1 fiber grouping were observed in one dog. Intramuscular nerve branches were mildly to moderately depleted of myelinated fibers. Large fiber loss was evident in the peroneal and vagus nerves resulting from axonal degeneration in two dogs. Regenerative changes were not obvious, and the vagosympathetic nerve did not reveal any specific abnormalities.

**Figure 3:**
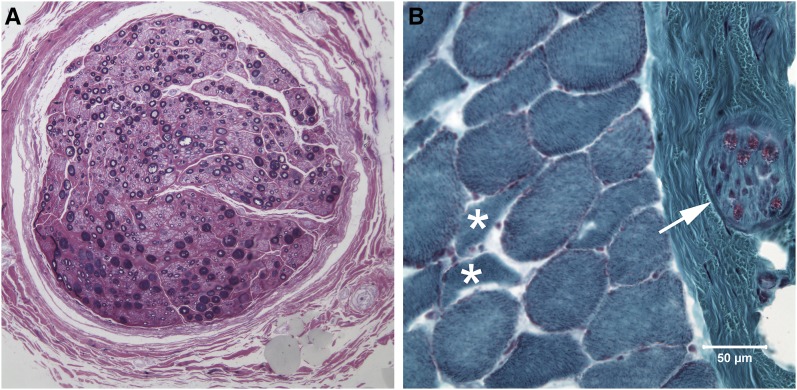
Peripheral nerve and muscle pathology. (A) Resin embedded 1 µm section of the peroneal nerve showing loss of large caliber myelinated nerve fibers without obvious regenerating clusters (toluidine blue stain). (B) Cryosection of the cranial tibial muscle showing atrophic fibers having an anguloid to angular shape (asterisks), and an intramuscular nerve branch that is moderately depleted of myelinated fibers (arrow). The pink stain within the intramuscular nerve branch localizes myelin in the individual nerve fibers (modified Gomori trichrome stain, bar = 50 µm for both A and B).

Large nerve fiber loss was evident in the peroneal nerve (Figure 3A), and vagus nerve resulting from chronic axonal degeneration. Regenerative changes were not obvious. No abnormalities were identified in the vagosympathetic nerve. In cryosections of the cranial tibial muscle, a mild variability in myofiber size was present, with scattered atrophic fibers having an angular to anguloid shape (Figure 3B), and of both fiber types. Fiber type grouping was not observed. Intramuscular nerve branches were moderately depleted of myelinated fibers.

### Genetic mapping of the causative genetic variant

The affected Alaskan Huskies did not have official pedigree certificates. However their breeder provided us with extensive pedigree information based on private records. The pedigrees were consistent with a monogenic autosomal recessive mode of inheritance, and it seemed likely that all affected Huskies used in this study were related.

At the time of the initial mapping of the causative locus we had samples from 18 closely related Alaskan Huskies, and one additional affected dog without close relatives (Figure S1). We performed parametric linkage analysis in the 18 related dogs, which consisted of four parents and 14 offspring (three affected/11 nonaffected). We obtained positive LOD scores for nine genome segments on eight chromosomes containing roughly 59.2 Mb in total. The highest LOD scores of 1.976 were obtained on chromosomes 2, 11, 15, 17, and 19 (Table S2).

Based on the pedigree records, we hypothesized that all affected dogs most likely were inbred to one single founder animal. Under this scenario, the affected individuals were expected to be identical by descent (IBD) for the causative mutation and flanking chromosomal segments. We therefore analyzed the four available cases for extended regions of homozygosity with simultaneous allele sharing, and found only a single genome region of 4.2 Mb that fulfilled our search criteria. The homozygous segment was on chromosome 19, and largely overlapped with one of the linked segments. The combined linkage and homozygosity analysis thus defined an exact critical interval of 4,086,630 bp at Chr19:36,483,638–40,570,267.

### Identification of the causative genetic variant

In order to obtain a comprehensive overview of all variants in the critical interval, we sequenced the genomes of one affected Alaskan Husky, one obligate carrier, and one unrelated purebred Siberian Husky at 13x–19x coverage. We called SNPs and indel variants with respect to the reference genome of a presumably nonaffected Boxer (CanFam 3.1). We additionally considered genome sequence data from 166 dog genomes of other breeds. Filtering for variants in the critical interval that were homozygous in the affected Alaskan Husky, heterozygous in the carrier, and absent from the unrelated Siberian Husky, and all the other 166 control genomes, resulted in only one perfectly disease-associated variant, chr19:36,535,487A > G. This SNP was intergenic and more than 30 kb away from the next annotated gene.

As this variant seemed unlikely to cause the severe observed phenotype, we then visually inspected the short read alignments in the critical interval to search for structural variants that would have most likely been missed by our automated variant detection pipeline. Several truncated read alignments in exon 7 of the *RAB3GAP1* gene indicated a potential duplication and/or insertion event (Figure S2).

**Figure 4:**
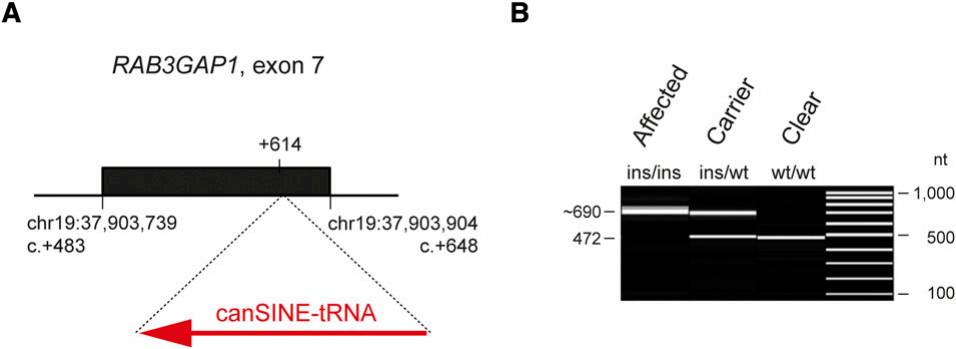
SINE insertion in exon 7 of the *RAB3GAP1* gene. (A) Schematic representation of the SINE insertion. A 218 bp canine SINE-tRNA insertion was found in affected Alaskan Huskies after position +614 of the *RAB3GAP1* coding sequence. Fourteen nucleotides flanking the insertion site were duplicated (File S2). (B) Experimental genotyping of the SINE insertion by fragment size analysis. We amplified exon 7 of the *RAB3GAP1* gene and flanking intron segments by PCR and separated the products of dogs with the three different genotypes by capillary gel electrophoresis. The size of the insertion allele might be slightly variable as the SINE insertion has a poly(A)-tract at its 3′-end. Such sequences are frequently not faithfully replicated, and thus show a high degree of length variation even between closely related individuals.

We designed primers flanking exon 7 and amplified this region in a long-range PCR. Sanger sequencing of the products revealed a 218 bp SINE insertion in exon 7 of the *RAB3GAP1* gene of the affected dogs (Figure 4). The insertion site was flanked by a 14 nucleotide duplication (File S2). The sequence of the mutant allele was submitted to the ENA under accession LN864704 and the mutant allele can be described as *RAB3GAP1:c.614_615insLN864704:g.123_340*.

We genotyped this variant by fragment length analysis in a larger cohort of 43 Alaskan Huskies, and 541 dogs of 68 diverse other breeds. The genotypes showed perfect cosegregation with the disease phenotype in Alaskan Huskies, and we did not find the SINE insertion outside of the Alaskan Husky population (Table 1).

**Table 1:** Association of the *RAB3GAP1* SINE insertion with affection status

Genotype	Alaskan Husky Cases*^a^*	Alaskan Husky Controls	Control Dogs from Other Breeds*^b^*
wt/wt	—	17	541
ins/ins	—	20	—
ins/ins	6	—	—

a Four cases had been available during the mapping phase of the project. Later, two affected maternal half siblings to an existing case became additionally available.

b A detailed list with breed affiliations of the control dogs can be found in Table S1. The SINE insertion was not found in Siberian Huskies (*n* = 2), Alaskan Malamutes (*n* = 6), Samoyedes (*n* = 25), or Greenland Dogs (*n* = 15).

### Analysis of the RAB3GAP1 transcript

**Figure 5:**
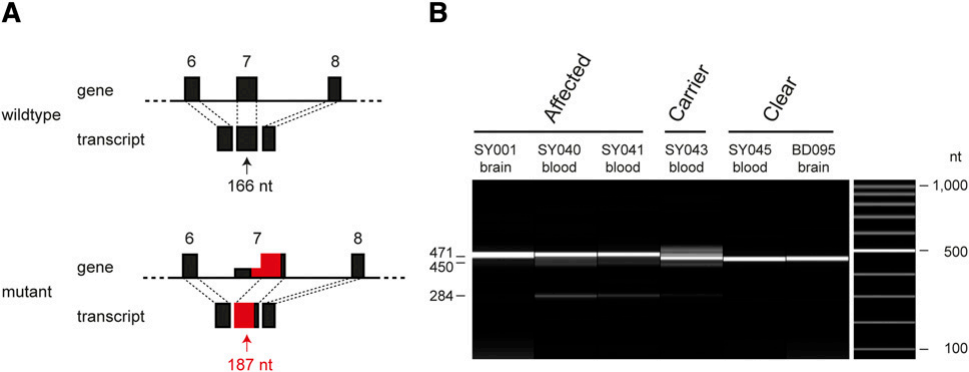
Effect of the SINE insertion on *RAB3GAP1* transcripts. (A) Schematic representation of exons 6 through 8 of the *RAB3GAP1* gene. Introns are not drawn to scale. The 218 bp SINE insertion into exon 7 is indicated in red. The SINE insertion leads to the utilization of a novel internal splice acceptor site and a mutant transcript, in which a large part of exon 7 is replaced with mutant sequence. In the mutant genomic allele the “intronized” parts of the original exon 7 are represented by a shallow rectangle. The new mutant exon 7 is indicated by a rectangle of the same height as exons 6 and 8. (B) Experimental confirmation of the aberrant alternative splicing. RT-PCR with primers in exon 6 and exon 8 amplified relatively uniform products of distinct sizes in dogs with the different genotypes. All RT-PCR products were Sanger sequenced to confirm their identity. In RNA from whole blood of affected animals, there is an additional faint band visible, which corresponds to a transcript lacking the entire exon 7.

We next investigated the effect of the 218 bp SINE insertion on the *RAB3GAP1* transcript, as the full-length insertion introduced several stop codons into the original exon 7. We isolated brain and blood RNA from dogs with the three different genotypes. RT-PCR with primers located in exons 6 and 8, and subsequent Sanger sequencing of the resulting products revealed that the SINE insertion led to aberrant splicing. In affected animals, the vast majority of the primary transcripts were spliced at a new internal splice acceptor site within the SINE insertion. This led to the inclusion of a novel “exon 7” containing 187 nucleotides instead of the wildtype exon 7 with 166 nucleotides (Figure 5). The mutant transcript preserved the original reading frame of the *RAB3GAP1* gene, and is predicted to encode a protein with 992 amino acids compared to the 985 amino acids of the wildtype canine RAB3GAP1. In the mutant protein, a stretch of 39 wildtype amino acids from positions 162–200 is replaced by 46 new amino acids with very little sequence homology (File S3).

With RNA from blood, but not with RNA from brain from affected dogs, we noticed a faint additional shorter RT-PCR product, which corresponded to a transcript lacking the entire exon 7.

Sanger sequencing of the RT-PCR products from a heterozygous carrier dog showed that transcripts from both the wildtype and the mutant allele were present at roughly similar amounts. Thus, nonsense-mediated decay (NMD) apparently is not a major consequence of the SINE insertion.

## Discussion

The observed clinical signs and pathological changes in the affected Alaskan Huskies closely resemble a phenotype originally described as “neuronal vacuolation and spinocerebellar degeneration” in Rottweilers (Kortz *et al.* 1997; Andrade-Neto *et al.* 1998; de Lahunta and Summers 1998; Eger *et al.* 1998; Pumarola *et al.* 1999). Similar phenotypes have also been reported in Boxers, and lately described in Black Russian Terriers as “polyneuropathy with ocular abnormalities and neuronal vacuolation (POANV)” (Geiger *et al.* 2009; Mhlanga-Mutangadura *et al.* 2014, 2015a, 2015b). While the underlying causative genetic variant in Boxers remains unknown, the causative genetic defect in Rottweilers and Black Russian Terriers has recently been identified by researchers from the University of Missouri in an independent study. Rottweilers and Black Russian Terriers with POANV were found to be homozygous for a single base deletion in the *RAB3GAP1* gene (c.743delC; Mhlanga-Mutangadura *et al.* 2014, 2015a, 2015b).

We have to caution here that our own genetic data reported in this study do not prove the causality of the identified *RAB3GAP1* SINE insertion for the Alaskan Husky disease on their own. Due to limited sample availability, our linkage analysis did not reach a LOD score of three, which is commonly accepted as the significance threshold to map a gene. We also did not confirm experimentally that the SINE insertion, and resulting aberrant splicing, really leads to a lack of functional RAB3GAP1 protein. However, many other known SINE insertions indeed completely abolish the function of the altered genes. Examples from dogs include centronuclear myopathy, and early retinal degeneration, which are caused by exonic SINE insertions into the *PTPLA* and *STK38L* genes, respectively (Pelé *et al.* 2005; Goldstein *et al.* 2010). We did not investigate whether the aberrant splicing in the Alaskan Huskies was due to actively regulated nonsense-associated altered splicing (Wang *et al.* 2002), or whether the SINE insertion just by chance contained a very strong splice acceptor motif, which redirects splicing to the new site. The SINE insertion seen in Labradors with centronuclear myopathy also leads to the utilization of the same internal splice acceptor site (Pelé *et al.* 2005).

Our genetic association data (Table 1), and the cosegregation of the SINE insertion with the POANV phenotype in the families, support the causality of the *RAB3GAP1* SINE insertion for the observed phenotype. The observed neuronal vacuolar changes are compatible with abnormal vesicular trafficking. The functional knowledge on the RAB3GAP complex from other species is consistent with this hypothesis. Finally, the fact that now two independent coding variants in the same gene have been found in dogs with an almost identical phenotype from independent dog populations, in our opinion establishes the causality of these variants beyond any reasonable doubt.

We found the *RAB3GAP1* SINE insertion only in Alaskan Huskies, but not in Siberian Huskies, Alaskan Malamutes, or any other closely related breed contributing to the Alaskan Husky gene pool. Based on the limited available pedigree information from the investigated cases, the hypothetical founder animal must have lived at least six generations before the investigated cases. As we tested only a very small number of Siberian Huskies and Alaskan Malamutes, these breeds should be monitored carefully to find whether they are truly free of the mutant *RAB3GAP1* allele.

The affected Alaskan Huskies show some similarities to human WARBM1. From a genetic point of view, human WARBM1 patients and the affected dogs both carry recessive loss-of-function alleles at the *RAB3GAP1* gene. The phenotype at first glance also shows some parallels with a combination of neurological abnormalities and ocular defects such as microphthalmia (Warburg *et al.* 1993; Aligianis *et al.* 2005; Morris-Rosendahl *et al.* 2010; Handley *et al.* 2013; Arroyo-Carrera *et al.* 2015). However, when studied in more detail, the human WARBM1 phenotype appears to be clinically more severe, and is characterized by a number of additional developmental defects that are not seen in the affected dogs, such as corpus callosum hypogenesis, or toe malformations.

Interestingly, *Rab3gap1* knockout mice show an even milder clinical phenotype than dogs. They appear grossly normal, with unremarkable eye and CNS development. The only reported phenotype is a reduced Ca^2+^-induced synaptic neurotransmitter release leading to alterations in the short-term plasticity of the hippocampal CA1 synapse (Sakane *et al.* 2006). It thus appears tempting to speculate that either the fine-tuning of synaptic vesicle release, which is mediated by the RAB3GAP complex, or a yet unknown additional function of the RAB3GAP complex somehow provides important clues for the correct development of the human brain. These hypothetical signals seem to be less important in dogs, and dispensable in mice. The *RAB3GAP1* mutant dogs thus provide an interesting intermediate phenotype, which might help to disentangle the various functions of the RAB3GAP complex.

In conclusion, we provide the first comprehensive description of a POANV phenotype in Alaskan Huskies, most likely caused by a SINE insertion in the *RAB3GAP1* gene. The affected dogs exhibit some parallels, but also clear phenotypic differences, with respect to human WARBM1 patients, and provide an animal model for the further functional analysis of the RAB3GAP complex. Our findings enable genetic testing in dogs, so that the nonintentional breeding of affected dogs can be avoided in the future.

## Supplementary Material

Supporting Information

## References

[bib1] Abecasis G. R. Cherny S. S. Cookson W. O. Cardon L. R., 2002 Merlin—rapid analysis of dense genetic maps using sparse gene flow trees. Nat. Genet. 30: 97–101. 11731797 10.1038/ng786

[bib2] Aligianis I. A. Johnson C. A. Gissen P. Chen D. Hampshire D., 2005 Mutations of the catalytic subunit of RAB3GAP cause Warburg Micro syndrome. Nat. Genet. 37: 221–223. 15696165 10.1038/ng1517

[bib3] Andrade-Neto J. P. Jardim L. S. Alessi A. C., 1998 Neuronal vacuolation in young Rottweilers. Vet. Rec. 143: 116. 9725182

[bib4] Arroyo-Carrera I. de Zaldívar Tristancho M. S. Bermejo-Sánchez E. Martínez-Fernández M. L. López-Lafuente A., 2015 Deletion 1q43–44 in a patient with clinical diagnosis of Warburg-Micro syndrome. Am. J. Med. Genet. A. 167: 1243–1251. 25899426 10.1002/ajmg.a.36878

[bib5] de Lahunta A. Summers B. A., 1998 The laryngeal lesion in young dogs with neuronal vacuolation and spinocerebellar degeneration. Vet. Pathol. 35: 316–317. 9684979 10.1177/030098589803500414

[bib6] Drögemüller M. Jagannathan V. Becker D. Drögemüller C. Schelling C., 2014 A mutation in the *FAM83G* gene in dogs with hereditary footpad hyperkeratosis (HFH). PLoS Genet. 10: e1004370. 24832243 10.1371/journal.pgen.1004370 PMC4022470

[bib7] Dubowitz V. Sewry C. A., 2013 Muscle biopsy: a practical approach, pp. 16–27 in Histological and Histochemical Stains and Reactions, Ed. 4, edited by Dubowitz V. Sewry C. A. Oldfors A. Saunders Elsevier, St. Louis.

[bib8] Eger C. E. Huxtable C. R. Chester Z. C. Summers B. A., 1998 Progressive tetraparesis and laryngeal paralysis in a young Rottweiler with neuronal vacuolation and axonal degeneration: an Australian case. Aust. Vet. J. 76: 733–737. 9862062 10.1111/j.1751-0813.1998.tb12301.x

[bib9] Fukui K. Sasaki T. Imazumi K. Matsuura Y. Nakanishi H., 1997 Isolation and characterization of a GTPase activating protein specific for the Rab3 subfamily of small G proteins. J. Biol. Chem. 272: 4655–4658. 9030515 10.1074/jbc.272.8.4655

[bib10] Geiger D. A. Miller A. D. Cutter-Schatzberg K. Shelton G. D. de Lahunta A., 2009 Encephalomyelopathy and polyneuropathy associated with neuronal vacuolation in two Boxer littermates. Vet. Pathol. 46: 1160–1165. 19605909 10.1354/vp.09-VP-0010-S-FL

[bib11] Goldstein O. Kukekova A. V. Aguirre G. D. Acland G. M., 2010 Exonic SINE insertion in STK38L causes canine early retinal degeneration (erd). Genomics 96: 362–368. 20887780 10.1016/j.ygeno.2010.09.003 PMC2996878

[bib12] Handley M. T. Morris-Rosendahl D. J. Brown S. Macdonald F. Hardy C., 2013 Mutation spectrum in RAB3GAP1, RAB3GAP2, and RAB18 and genotype-phenotype correlations in Warburg micro syndrome and Martsolf syndrome. Hum. Mutat. 34: 686–696. 23420520 10.1002/humu.22296

[bib13] Kortz G. D. Meier W. A. Higgins R. J. French R. A. McKiernan B. C., 1997 Neuronal vacuolation and spinocerebellar degeneration in young Rottweiler dogs. Vet. Pathol. 34: 296–302. 9240838 10.1177/030098589703400405

[bib14] Liegel R. P. Handley M. T. Ronchetti A. Brown S. Langemeyer L., 2013 Loss-of-function mutations in TBC1D20 cause cataracts and male infertility in blind sterile mice and Warburg micro syndrome in humans. Am. J. Hum. Genet. 93: 1001–1014. 24239381 10.1016/j.ajhg.2013.10.011 PMC3852926

[bib15] Mhlanga-Mutangadura, T., G. S. Johnson, G. C. Johnson, L. Hansen, G. V. Tamassia *et al.*, 2014 The whole genome sequences from a Rottweiler and Black Russian Terrier with overlapping neurological syndromes contain the same *RAB3GAP1* frame shift mutation. Conference abstract no. 31 at the Advancing Neuroscience at MU Symposium. Available at: http://medicine.missouri.edu/conferences/mu-neuroscience/MU%20Neuroscience%20Symposium%20Abstracts.pdf.

[bib16] Mhlanga-Mutangadura, T., G. S. Johnson, G. C. Johnson, L. Hansen, G. V. Tamassia *et al.*, 2015a A homozygous RAB3GAP1:c.743delC deletion in Black Russian terriers with laryngeal paralysis, polyneuropathy, ocular abnormalities and neuronal vacuolation. Abstract at the 8th International Conference on Advances in Canine and Feline Genomics and Inherited Disease. Available at: http://www.caninefelinegenomicsconference.org/wp-content/uploads/2014/11/AHTConference2015.pdf.

[bib17] Mhlanga-Mutangadura T. Johnson G. S. Schnabel R. D. Taylor J. F. Johnson G. C., 2015 b A mutation in the Warburg syndrome gene, RAB3GAP1, causes a similar syndrome with polyneuropathy and neuronal vacuolation in Black Russian Terrier dogs. Neurobiol Dis. *Neurobiology of Disease* doi:10.1016/j.nbd.2015.11.016. 10.1016/j.nbd.2015.11.016 26607784

[bib18] Morris-Rosendahl D. J. Segel R. Born A. P. Conrad C. Loeys B., 2010 New RAB3GAP1 mutations in patients with Warburg Micro Syndrome from different ethnic backgrounds and a possible founder effect in the Danish. Eur. J. Hum. Genet. 18: 1100–1106. 20512159 10.1038/ejhg.2010.79 PMC2987448

[bib19] Oishi H. Sasaki T. Nagano F. Ikeda W. Ohya T., 1998 Localization of the Rab3 small G protein regulators in nerve terminals and their involvement in Ca2+-dependent exocytosis. J. Biol. Chem. 273: 34580–34585. 9852129 10.1074/jbc.273.51.34580

[bib20] Pelé M. Tiret L. Kessler J. L. Blot S. Panthier J. J., 2005 SINE exonic insertion in the *PTPLA* gene leads to multiple splicing defects and segregates with the autosomal recessive centronuclear myopathy in dogs. Hum. Mol. Genet. 14: 1417–1427. 15829503 10.1093/hmg/ddi151

[bib21] Pumarola M. Fondevila D. Borrás D. Majó N. Ferrer I., 1999 Neuronal vacuolation in young Rottweiler dogs. Acta Neuropathol. 97: 192–195. 9928831 10.1007/s004010050973

[bib22] Purcell S. Neale B. Todd-Brown K. Thomas L. Ferreira M. A., 2007 PLINK: a tool set for whole-genome association and population-based linkage analyses. Am. J. Hum. Genet. 81: 559–575. 17701901 10.1086/519795 PMC1950838

[bib23] Sakane A. Manabe S. Ishizaki H. Tanaka-Okamoto M. Kiyokage E., 2006 Rab3 GTPase-activating protein regulates synaptic transmission and plasticity through the inactivation of Rab3. Proc. Natl. Acad. Sci. USA 103: 10029–10034. 16782817 10.1073/pnas.0600304103 PMC1502500

[bib24] Seuberlich T. Botteron C. Benestad S. L. Brünisholz H. Wyss R., 2007 Atypical scrapie in a Swiss goat and implications for transmissible spongiform encephalopathy surveillance. J. Vet. Diagn. Invest. 19: 2–8. 17459826 10.1177/104063870701900102

[bib26] Wang J. Chang Y. F. Hamilton J. I. Wilkinson M. F., 2002 Nonsense-associated altered splicing: a frame-dependent response distinct from nonsense-mediated decay. Mol. Cell 10: 951–957. 12419238 10.1016/s1097-2765(02)00635-4

[bib27] Warburg M., O. Sjö, H. C. Fledelius, and S. A. Pedersen, 1993 Autosomal recessive microcephaly, microcornea, congenital cataract, mental retardation, optic atrophy, and hypogenitalism. Micro syndrome. Am. J. Dis. Child. 147: 1309–1312. 8249951 10.1001/archpedi.1993.02160360051017

