# Supplementary material for: A RAB3GAP1 SINE Insertion in Alaskan Huskies with Polyneuropathy, Ocular Abnormalities, and Neuronal Vacuolation (POANV) Resembling Human Warburg Micro Syndrome 1 (WARBM1)
Source: G3 (Bethesda). 2015 Nov 23;6(2):255–62. doi: 10.1534/g3.115.022707 (PMC4751546; doi:10.1534/g3.115.022707)
Supplement: Supporting Information [file supp_6_2_255__index.html]

A RAB3GAP1 SINE Insertion in Alaskan Huskies with Polyneuropathy, Ocular Abnormalities, and Neuronal Vacuolation (POANV) Resembling Human Warburg Micro Syndrome 1 (WARBM1) — Supporting Information 

# A *RAB3GAP1* SINE Insertion in Alaskan Huskies with Polyneuropathy, Ocular Abnormalities, and Neuronal Vacuolation (POANV) Resembling Human Warburg Micro Syndrome 1 (WARBM1)

## Supporting Information for Wiedmer *et al.*, 2016

**Files in this Data Supplement:**

- File S2 - Sequence context of the 218 bp SINE insertion into exon 7 of the canine *RAB3GAP1* gene. (.pdf, 132 KB)
- File S3 - Alignment of the canine wildtype and predicted mutant RAB3GAP1 protein. (.pdf, 45 KB)
- Figure S1 - Pedigrees of Alaskan Huskies used for the mapping of the disease locus. (.pdf, 303 KB)
- Figure S2 - IGV screenshot of the region with the SINE insertion. (.pdf, 155 KB)
- File S1 - Video of an affected Alaskan Husky (SY005) at 8 months of age. (.wmv, 9,403 KB)
- Table S1 - *RAB3GAP1*:c.614\_615ins218 genotypes of 541 dogs from 68 different dog breeds. (.xlsx, 15 KB)
- Table S2 - Genome regions that showed positive LOD scores in the linkage analysis. (.xlsx, 14 KB)
