# Supplementary material for: A RAB3GAP1 SINE Insertion in Alaskan Huskies with Polyneuropathy, Ocular Abnormalities, and Neuronal Vacuolation (POANV) Resembling Human Warburg Micro Syndrome 1 (WARBM1)
Source: G3 (Bethesda). 2015 Nov 23;6(2):255–62. doi: 10.1534/g3.115.022707 (PMC4751546; doi:10.1534/g3.115.022707)
Supplement: Supporting Information [file supp_g3.115.022707_FileS2.pdf]

File S1: Sequence context of the 218 bp SINE insertion into exon 7 of the canine *RAB3GAP1* gene

Genetic variant:	Chr19:37,903,870_37,903,871ins218	(CanFam 3.1 assembly)
	RAB3GAP1:c.614_615ins218	(XM_851254.3)
	RAB3GAP1:c.614_615insLN864704:g.123_340	(XM_851254.3)

CanFam 3.1, chromosome 31 genomic sequence, positions 37,903,701 – 37,904,200

218 bp SINE/tRNA-insertion in red

Wildtype *RAB3GAP1* exon 7, 166 nucleotides in black CAPITAL LETTERS (166 nt)

Mutant *RAB3GAP1* exon 7, arising from the usage of an internal cryptic splice site is underlined (187 nt)

```
37,903,701      gaacttaaag tccattccca tttaattgtg tcctacagCC AGGTGCCACT CTTTGTGCAA   37,903,760
                          >>>>>>>>>>>>>>>>>>>>>>>>>>>>

37,903,761      ATTCATCATA AATGGCGAAG AATGTATGTG GGCGAATGTC AGGGTCATGG TGTCCGAACT   37,903,820
37,903,821      GATTTTGAAA TGGTTCATCT TCGGAAAGTG CCAAATCAGT ACACTCATTT              37,903,870
                                                     14 nt duplication flanking the insertion

37,903,870.1    TTTTTTTTTT TTTTTTTTTT TTTTTAATAT TTTTATTTTT TTTAATTTTT ATTTATTTAT   37,903,870.60
37,903,870.61   GATAGTTACA GAGAGAGAGA GAGGCAGAGA CACAGGCAGA GGGAGAAGCA GGCTCCATGC   37,903,870.120
37,903,870.121  ACCGGGAGCC TGACGTGGGA TTCGATCCCG GGTCTCCAGG ATCGCGCCCT GGGCCAAAGG   37,903,870.180
37,903,870.181  CAGGCACCAA ACCGCTGCGC CACCCAGTAC ACTCATTT                          37,903,870.218
                                          14 nt duplication flanking the insertion

37,903,871                                                             ATCAGGTCTG   37,903,880
37,903,881      CTGGATATCT TCAAATCAAA GATTgtgagt ttgcattgat attgtcaatc ttatctggga   37,903,940
37,903,941      tccagttaaa aatagcaatt tagtaatttt attgagtatg tagaaacatt tctctgtaat   37,904,000
37,904,001      attcatcatg taattagttg gaaggaagat ataattgact acataaatta ctatgtctgt   37,904,060
37,904,061      ttaaagaaac agtatctagt tagattttta gtgtactaat gctaaaaaaa caattaaact   37,904,120
37,904,121      aatattgttc aatttgttaa gatttattta ctgtgttatc tcttcctact ccttttcctc   37,904,180
                                                  <<<<<<<<<<<<<<<<<<<<<<<<
37,904,181      taagttgacc cttaaacatc                                               37,904,200
                <
```

PCR primers for the amplification of a genomic 472 bp product (wildtype allele) vs. a genomic 690 bp product (mutant allele) are indicated beneath the sequence with “>>>” and “<<<”.
