# Supplementary material for: A RAB3GAP1 SINE Insertion in Alaskan Huskies with Polyneuropathy, Ocular Abnormalities, and Neuronal Vacuolation (POANV) Resembling Human Warburg Micro Syndrome 1 (WARBM1)
Source: G3 (Bethesda). 2015 Nov 23;6(2):255–62. doi: 10.1534/g3.115.022707 (PMC4751546; doi:10.1534/g3.115.022707)
Supplement: Supporting Information [file supp_g3.115.022707_FileS3.pdf]

**File S3: Alignment of the canine wildtype and predicted mutant RAB3GAP1 protein**

```
Wildtype  1    MAADSEPESEVFEITDFTTASEWERFISKVEEVLNDWKLIGNSLGKPLEKGIFTSGAWEE  60
               ||||||||||||||||||||||||||||||||||||||||||||||||||||||||||||
Mutant    1    MAADSEPESEVFEITDFTTASEWERFISKVEEVLNDWKLIGNSLGKPLEKGIFTSGAWEE  60

Wildtype  61   KSDEISFADFKFSVTHHYLVQDSADKEGKDEVLEDVIPQSMQDLLCMNNDFPPRAHCLVR  120
               ||||||||||||||||||||||||||||||||||||||||||||||||||||||||||||
Mutant    61   KSDEISFADFKFSVTHHYLVQDSADKEGKDEVLEDVIPQSMQDLLCMNNDFPPRAHCLVR  120

Wildtype  121  WYGLREFVVIAPAANHDAVLSESKCNLLLSSVSIALGNTGCQVPLFVQIHHKWRRMYVGE  180
               |||||||||||||||||||||||||||||||||||||||||      | | :  |
Mutant    121  WYGLREFVVIAPAANHDAVLSESKCNLLLSSVSIALGNTGCYRERERQRHRQ--REKQAP  178

Wildtype  181  CQGHGVRTDF-----EMVHLRKVPN----QYTHLSGLLDIFKSKIGCPLTPLPPVSIAIR  231
               | |              :  |: ||    |||||||||||||||||||||||||||||||
Mutant    179  CTGSLTWDSIPGLQDRALGQRQAPNRCATQYTHLSGLLDIFKSKIGCPLTPLPPVSIAIR  238

Wildtype  232  FTYVLQDWQQYFWPQQPPDIDALVGGEVGGLEFGKLPFGACEDPISELHLATTWPHLTEG  291
               ||||||||||||||||||||||||||||||||||||||||||||||||||||||||||||
Mutant    239  FTYVLQDWQQYFWPQQPPDIDALVGGEVGGLEFGKLPFGACEDPISELHLATTWPHLTEG  298

Wildtype  292  IIVDNDVYSDLDPIQAPHWSVRVRKADNPQCLLGDFVTEFFKICRRKESTDEILGRSTFE  351
               ||||||||||||||||||||||||||||||||||||||||||||||||||||||||||||
Mutant    299  IIVDNDVYSDLDPIQAPHWSVRVRKADNPQCLLGDFVTEFFKICRRKESTDEILGRSTFE  358

Wildtype  352  EEGREIADITHALSKLTEPAPVPIHKLSVSNMVHTAKKKIRKHRGVEESPLNNDVLNTIL  411
               ||||||||||||||||||||||||||||||||||||||||||||||||||||||||||||
Mutant    359  EEGREIADITHALSKLTEPAPVPIHKLSVSNMVHTAKKKIRKHRGVEESPLNNDVLNTIL  418

Wildtype  412  LFLFPDAASEKPLDGSSSTDSNNPLSESEEYNLYNQFKSAPSDSLTYKLALCLCMINFYH  471
               ||||||||||||||||||||||||||||||||||||||||||||||||||||||||||||
Mutant    419  LFLFPDAASEKPLDGSSSTDSNNPLSESEEYNLYNQFKSAPSDSLTYKLALCLCMINFYH  478

Wildtype  472  GGLKGVAHLWQEFVLEMRFRWENNFLIPGLTSGPPDLRCCLLHQKLQMLNCCIERKKARD  531
               ||||||||||||||||||||||||||||||||||||||||||||||||||||||||||||
Mutant    479  GGLKGVAHLWQEFVLEMRFRWENNFLIPGLTSGPPDLRCCLLHQKLQMLNCCIERKKARD  538

Wildtype  532  EGRKTNTSEIYPGDTGKAGDQLGPDNLKDMDKEKGEIGKSWDSWSDSEEEFFECLSDTEE  591
               ||||||||||||||||||||||||||||||||||||||||||||||||||||||||||||
Mutant    539  EGRKTNTSEIYPGDTGKAGDQLGPDNLKDMDKEKGEIGKSWDSWSDSEEEFFECLSDTEE  598

Wildtype  592  LKGNGQESGKKGGPKEVASLKPEGRLHQHGKLTLLHNGEPLYIPVTQEPAPMTEDLLEEQ  651
               ||||||||||||||||||||||||||||||||||||||||||||||||||||||||||||
Mutant    599  LKGNGQESGKKGGPKEVASLKPEGRLHQHGKLTLLHNGEPLYIPVTQEPAPMTEDLLEEQ  658

Wildtype  652  SEVLAKLGTSAEGAHLRARMQSACLLSDMESFKAANPGCFLEDFVRWYSPRDYIEEEVVD  711
               ||||||||||||||||||||||||||||||||||||||||||||||||||||||||||||
Mutant    659  SEVLAKLGTSAEGAHLRARMQSACLLSDMESFKAANPGCFLEDFVRWYSPRDYIEEEVVD  718

Wildtype  712  EKGNMVLKGELSARMKIPSNMWVEAWETAKPIPARRQRRLFDDTREAEKVLHYLAVQKPA  771
               ||||||||||||||||||||||||||||||||||||||||||||||||||||||||||||
Mutant    719  EKGNMVLKGELSARMKIPSNMWVEAWETAKPIPARRQRRLFDDTREAEKVLHYLAVQKPA  778

Wildtype  772  DLARHLLPCVIHAAVLKVKEEENLENISSVKKIIKQIITHSSKVLHFPNPEDKKLEEIIH  831
               ||||||||||||||||||||||||||||||||||||||||||||||||||||||||||||
Mutant    779  DLARHLLPCVIHAAVLKVKEEENLENISSVKKIIKQIITHSSKVLHFPNPEDKKLEEIIH  838

Wildtype  832  QITNVEAIIARARSLKAKFGTEKCEQEEEKEDLERFVSCLLEQPEVLVVGAGRGHAGKII  891
               ||||||||||||||||||||||||||||||||||||||||||||||||||||||||||||
Mutant    839  QITNVEAIIARARSLKAKFGTEKCEQEEEKEDLERFVSCLLEQPEVLVVGAGRGHAGKII  898

Wildtype  892  HKLFVNAQRLTESSDEAAAVAPPEEELKRMGSPEERRHNSVSDFPPPAGRELILRTTVPR  951
               ||||||||||||||||||||||||||||||||||||||||||||||||||||||||||||
Mutant    899  HKLFVNAQRLTESSDEAAAVAPPEEELKRMGSPEERRHNSVSDFPPPAGRELILRTTVPR  958

Wildtype  952  PAPYSRALPQRMYSVLTKEDFRLAGAFSSDTTFF  985
               ||||||||||||||||||||||||||||||||||
Mutant    959  PAPYSRALPQRMYSVLTKEDFRLAGAFSSDTTFF  992
```
