# Supplementary material for: A RAB3GAP1 SINE Insertion in Alaskan Huskies with Polyneuropathy, Ocular Abnormalities, and Neuronal Vacuolation (POANV) Resembling Human Warburg Micro Syndrome 1 (WARBM1)
Source: G3 (Bethesda). 2015 Nov 23;6(2):255–62. doi: 10.1534/g3.115.022707 (PMC4751546; doi:10.1534/g3.115.022707)
Supplement: Supporting Information [file supp_g3.115.022707_FigureS1.pdf]

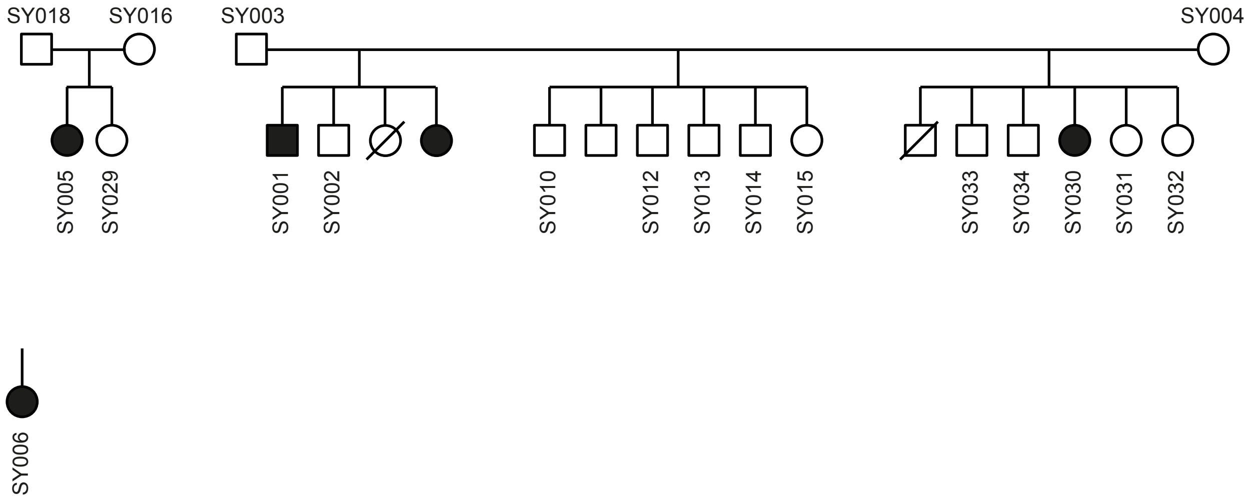

Figure S1 Pedigrees of Huskies used for the mapping of the disease locus. All dogs originated from a single breeder and were assumed to trace back to a recent common ancestor. Filled symbols represent affected animals. Two dogs died young without a neurological examination and may also have been affected (indicated by strike-through symbols). Numbers indicate dogs, which were used for linkage and/or homozygosity mapping. We thus had 28 infomative meioses for linkage mapping and 4 affected dogs for homozygosity mapping.
