# Supplementary material for: A RAB3GAP1 SINE Insertion in Alaskan Huskies with Polyneuropathy, Ocular Abnormalities, and Neuronal Vacuolation (POANV) Resembling Human Warburg Micro Syndrome 1 (WARBM1)
Source: G3 (Bethesda). 2015 Nov 23;6(2):255–62. doi: 10.1534/g3.115.022707 (PMC4751546; doi:10.1534/g3.115.022707)
Supplement: Supporting Information [file supp_g3.115.022707_FigureS2.pdf]

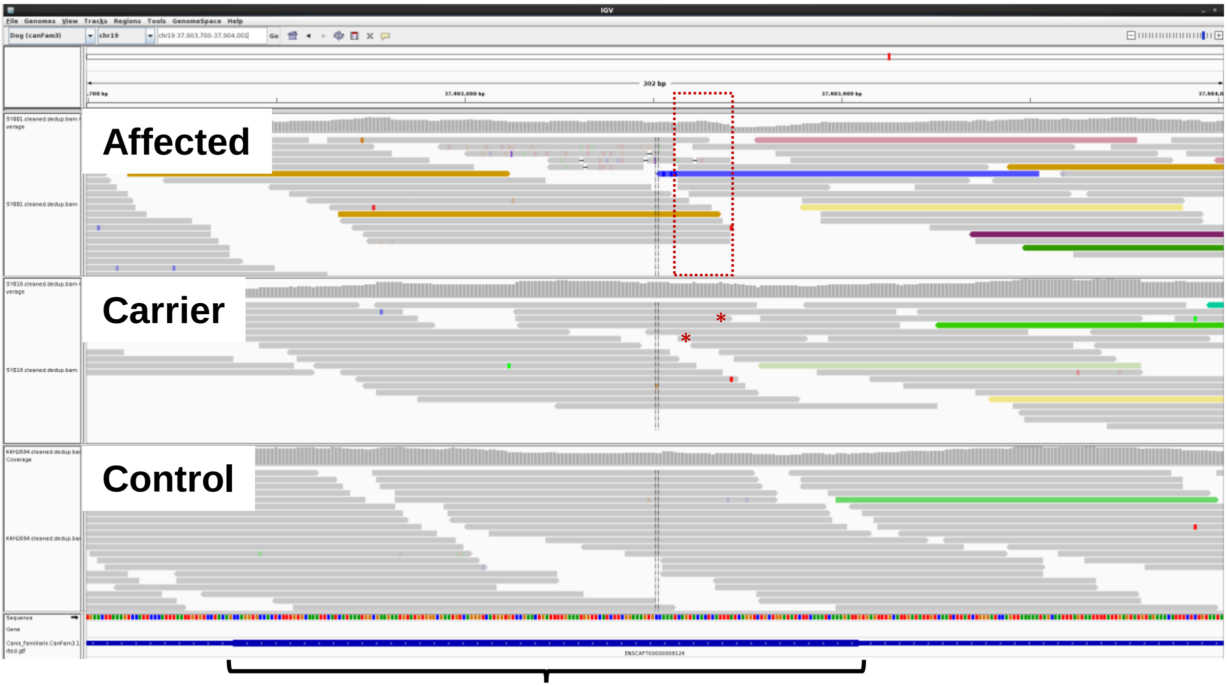


*RAB3GAP1*, exon 7

**Figure S2** IGV screenshot of the region with the SINE insertion. The sequence alignments of the affected Husky (SY001), a heterozygous obligate carrier (SY018), and an unrelated purebred Siberian Husky (KKH2684) are displayed. The dashed red box in the affected dog indicates a region where all forward and reverse read alignments are truncated. The truncated reads overlap by 14 nucleotides, which is caused by the duplication of 14 nucleotides flanking the insertion site of the SINE. In the carrier animal two similarly truncated read alignments are indicated by red asterisks. The control animal shows normal overlapping alignments of the 100 bp illumina reads with respect to the reference sequence. The insertion site is within exon 7 of the *RAB3GAP1* gene.
